# Hospital Meetings, &c.

**Published:** 1899-07-22

**Authors:** 


### HOSPITAL MEETINGS, &C.

### LEGISLATION FOR MIDWIVES.

The supporters of the principle of regulating the practice of midwives by legislation had an interview with the Duke of Devonshire, at the Privy Council, Whitehall, on Tuesday, with the view of getting the Government to take up in the next session of Parliament the Bill for the compulsory registration of midwives. Among those present were the Duke of Westminster, Mr. Heywood Johnstone, M.P., Sir J. Colomb, M.P., Mr. C. Schwann, M.P., Mr. Hazell, M.P., Mr. H. J. Tennant, M.P., Lady Balfour of Burleigh, Mrs. Sheldon Amos, the Hon. Mrs. Alfred Lyttelton, and representatives of the London Obstetrical Society, Queen Victoria's Jubilee Institute, Women's Liberal Federation, Women's National Liberal Association, Women's Liberal Unionist Association, Women's Co-operative Guild, Women's Industrial Council, National Union of Women Workers, and the Midwives' Institute. The Duke of Devonshire was accompanied by Sir Richard Thorne.

Mr. Heywood Johnstone, in introducing the subject, said the need of some legislative provision for the training of midwives and the regulation of their practice had been recognised for a good many years past. The General Medical Council had reported several times that measures should be taken to protect mothers of the poorer classes from the terrible risks which they and their children now run through the work of ignorant midwives. His Grace advised a deputation which waited upon him last year to ascertain the views and wishes of the General Medical Council, to ascertain the views of the local authorities who would have to carry out the Act, and also to consider the question of cost. They had had a conference with the General Medical Council, and the Bill of this session embodied the recommendations of the Medical Council. The Royal College of Physicians and the Royal College of Surgeons of England had also considered the Bill, and they had come to a general agreement on the main lines on which legislation should proceed—viz., to obtain qualified midwives who would be under local supervision, and for the prohibition of unqualified practitioners. The County Councils' Association had also approved of the general principles. Everyone was agreed as to the necessity of a Central Board to frame rules and regulations to carry out the Act, but when it came to the position of that Central Board there were varying views, and they recognised that the Government must have the settlement of it. Having had the advantage of a conference with the General Medical Council, as representing the informed medical opinion of the country, and having also brought the matter before the County Councils' Association, and the principles being practically agreed upon, the details only remained to be settled by the Government. They therefore asked his Grace to lay the matter before the Cabinet, with the view of its being taken up in the next Session of Parliament.

The Duke of Devonshire: I understand you to say that you have been in communication with the General Medical Council, and that you have come to an agreement; but I have the report of the committee of the General Medical Council, dated May 30th, 1899, in which they make a great many criticisms and conclude as follows: "The committee accordingly recommend the Council to inform the Lord President of the Privy Council that they are unable to approve the Bill unless it is recast in accordance with the suggestions contained in his report."

Mr. Heywood Johnstone: But these are matters of detail which do not affect the principle of the Bill. The only reason why we have not recast the Bill is because they are matters which should be dealt with by the Government.

Mr. C. J. Cullingworth, M.D., said the General Medical Council's amendments were matters of detail to which the deputation had no objection.

Mr. Bezley Thorne, representing the Royal British Nurses' Association, Dr. George Rice, medical officer of health for Derby, Mr. Will Crookes, L.C.C., and Mr. George Roumieu, the president of the Coroners' Society for England and Wales, endorsed what had been said by previous speakers.

The Duke of Devonshire, in reply, said that the statements which had been made should receive his most careful consideration, and after encouraging the deputation to proceed with their work, said: "I can promise you that I will bring the matter as fully and fairly as I can before my colleagues with the view of inducing them to take the matter into their practical consideration. Whether the Government is able to deal with the matter or not next session, I would suggest that you should make use of this department, of which I am the head, as the means of communication with those authorities whose support it is necessary for you to enlist—namely, the medical profession and the local authorities. Any assistance we can give to you in arriving at a complete and perfect understanding with them we shall be very happy to render, and it is quite possible that, even if the Government should not be able to devote the necessary amount of time to the measure, considerable progress may be made by the introduction of a Bill by a private member.

Mr. Heywood Johnstone thanked his Grace for his encouraging words, which, he said, marked a decided progress in the matter; and the deputation withdrew.

### THE LONDON HOSPITAL.

### Opening of New College Buildings.

On Tuesday afternoon the most recent additions to the Medical College of the London Hospital were opened by Lord Knutsford, and prizes distributed to the students and probationers. Lord and Lady Knutsford arrived a little after four o'clock, and were received by Mr. Douro Hoare (chairman of the College Board), the Hon. Sydney Holland (chairman of the hospital), and the members of the House Committee and medical staff; proceeding, after a visit to the new buildings, to the library, where they were awaited by a large audience. Amongst those who accepted an invitation to be present were Viscount and Viscountess Raincliffe, the Hon. Evelina Rothschild, Lady Beatrice Herbert, the Hon. Edith Boscawen, Sir W. Pearson, Sir Fowell Buxton, Sir Henry Burdett, Mr. T. Spender, &c., &c. On the platform were the principal guests: Mr. Holland and Mr. Hoare, Dr. Stephen Mackenzie (senior physician), Mr. Frederick Treves (consulting surgeon), and other members of the College Board and Hospital Committee.

Mr. Douro Hoare having opened the proceedings, Lord Knutsford declared the new buildings open. He spoke of the recent addition to the Hospital Medical College as essential to the welfare of the institution, and therefore to that of the working population of the East-end of London. Those last four words meant a large area covered with houses in which thousands of men, women, and children were packed together. It was certain that where people were closely packed together there must be a vast amount of illness, and for this seething population the London Hospital provided the only general help in that enormous neighbourhood. The Poplar Hospital dealt in an admirable manner with accidents, but had not yet begun to treat disease. Many people were no doubt acquainted with the noble work of the London Hospital, but fewer realised how intimately it was connected with that of the Medical College. In recent years great strides had been made in medical knowledge, and it was most satisfactory to know that the London Hospital Medical College was in the proud position of having the best equipped bacteriology department in the Metropolis. He had great pleasure in declaring the new building open.

The presentation of prizes to the students followed, after which Mr. Holland gave some account of the work of the hospital and of the improvements and alterations now being made, appealing for the financial help which was needed to carry out the scheme proposed. Lady Knutsford presented the prizes to the probationers, and votes of thanks to Lord and Lady Knutsford were proposed by Dr. Stephen Mackenzie, and seconded in an admirable speech by Mr. Treves. After the ceremony the college, hospital, and nurses' home were thrown open for general inspection by the visitors, and tea was served in the garden.

The new block of the college stands on the site of the old chemical theatre and laboratory, and contains in the basement a department of public health, museum, professors' and class rooms; on the first floor, clinical theatre and laboratories, and balance room. Above are physics and chemical laboratories, operative surgery room, and anatomy class room, and on the third floor the bacteriological department, with general and research laboratories, sterilising room, and more class rooms. Additions have been also made in other departments, and the London Hospital has now good reason to be proud of its medical college. The new buildings, with their fittings, are estimated to have cost not less than £10,000.

